# Multi-arm RNA junctions encoding molecular logic unconstrained by input sequence for versatile cell-free diagnostics

**DOI:** 10.1038/s41551-022-00857-7

**Published:** 2022-03-14

**Authors:** Duo Ma, Yuexin Li, Kaiyue Wu, Zhaoqing Yan, Anli A. Tang, Soma Chaudhary, Zachary M. Ticktin, Jonathan Alcantar-Fernandez, José L. Moreno-Camacho, Abraham Campos-Romero, Alexander A. Green

**Affiliations:** 1 Biodesign Center for Molecular Design and Biomimetics at the Biodesign Institute, Arizona State University, Tempe, AZ USA; 2 Department of Biomedical Engineering, Boston University, Boston, MA USA; 3 Molecular Biology, Cell Biology and Biochemistry Program, Graduate School of Arts and Sciences, Boston University, Boston, MA USA; 4 Clinical Laboratory Department, Salud Digna, Culiacan, Sinaloa, Mexico; 5 Biological Design Center, Boston University, Boston, MA USA; 6 Present Address: BeiGene (Shanghai) Co., Ltd., Shanghai, China

**Keywords:** Biosensors, Assay systems, RNA, Synthetic biology

## Abstract

Applications of RNA-based molecular logic have been hampered by sequence constraints imposed on the input and output of the circuits. Here we show that the sequence constraints can be substantially reduced by appropriately encoded multi-arm junctions of single-stranded RNA structures. To conditionally activate RNA translation, we integrated multi-arm junctions, self-assembled upstream of a regulated gene and designed to unfold sequentially in response to different RNA inputs, with motifs of loop-initiated RNA activators that function independently of the sequence of the input RNAs and that reduce interference with the output gene. We used the integrated RNA system and sequence-independent input RNAs to execute two-input and three-input OR and AND logic in *Escherichia coli*, and designed paper-based cell-free colourimetric assays that accurately identified two human immunodeficiency virus (HIV) subtypes (by executing OR logic) in amplified synthetic HIV RNA as well as severe acute respiratory syndrome coronavirus-2 (via two-input AND logic) in amplified RNA from saliva samples. The sequence-independent molecular logic enabled by the integration of multi-arm junction RNAs with motifs for loop-initiated RNA activators may be broadly applicable in biotechnology.

## Abstract (web summary)

Multi-arm junction RNAs integrating motifs for loop-initiated RNA activators enable the execution of molecular logic independent of RNA-input sequence, thus facilitating the design of cell-free diagnostics.

## Main

RNA adopts diverse secondary and tertiary structures that enable it to perform a variety of different roles in the cell, from regulating gene expression and catalysing chemical reactions to sensing small molecules and scaffolding proteins^1–5^. RNA molecules designed to fold into diverse secondary structures have been used to tightly regulate gene expression at the transcriptional and translational levels in response to trans-acting RNAs^6,7^, small molecules^8^, proteins^9^, or specified logic expressions^10,11^, and have been used in strand-displacement systems for computing and imaging applications^12,13^. Moreover, they have found use in low-cost systems for detection of viruses^14–16^, mutations^17^, and for water testing^18,19^. At the same time, the structural diversity of RNA has been harnessed in RNA nanotechnology to generate a variety of RNA-based nanostructures with complex geometries through self-assembly^20–22^. These structures are assembled from molecular building blocks featuring hairpins, multi-arm junctions, and other structural elements programmed to fold into prescribed structures through combinations of dangling end, kissing loop, and crossover interactions^23–27^. Such assemblies have enabled the production of multivalent nanoparticles carrying siRNA payloads^28–30^ and have also been synthesized within living cells enabling enzyme localization^31,32^. They provide a wealth of different RNA structures that can be harnessed for programming cellular function.

**Extended Data Fig. 1:**
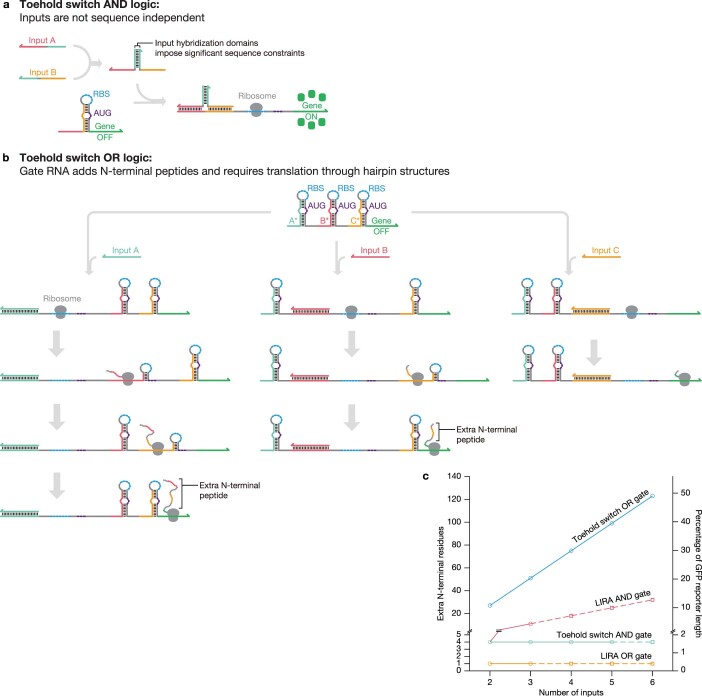
Limitations of previously reported toehold-switch-based logic systems. **a**, Toehold-mediated two-input AND logic requires hybridization between input RNAs to activate gene expression. This requirement is challenging to meet when detecting natural RNA sequences, such as those from viruses. **b**, Toehold-mediated three-input OR gates consist of three toehold switches assembled one after the other in the same open reading frame. This design appends extended N-terminal peptides to the output protein depending on the input species binding site. The ribosome must also translate through downstream toehold switch hairpins, which leads to substantial variations in output protein expression levels. **c**, Scaling of the largest reporter protein N-terminal peptide generated as a function of gate RNA architecture and number of inputs. Toehold-mediated OR gate RNAs append a peptide of up to 49% of the length of GFP for six-input logic. LIRA AND gates also generate N-terminal peptides, but peptide size scales at a much lower rate. Circles mark gate RNAs that have been demonstrated experimentally and squares are projections for gate RNAs that have yet to be demonstrated experimentally.

Taking concepts from RNA nanotechnology and RNA-based regulation of gene expression^33,34^, recent years have seen the development of self-assembly-driven molecular computing systems that operate in living cells and exploit the combined interactions of multiple carefully designed synthetic RNAs^11,35–37^. Such ribocomputing devices act by modulating gene expression in response to specified combinations of input RNAs and take advantage of the predictability of RNA–RNA interactions to enable effective computer-based design. These systems have been used to carry out combinations of AND, OR, NAND and NOR logic with up to a dozen inputs and have operated using complexes formed from as many as five distinct RNAs in living cells^11,36^. The ribocomputing devices developed thus far, however, have had several critical limitations that constrain the range of input RNAs that they can monitor and the range of output proteins that they can produce. These systems have relied on hybridization between multiple input RNAs for implementing AND logic, limiting their use against natural transcripts that lack the necessary complementarity between sequences or requiring adapter strands that reduce system output (Extended Data Fig. 1a). Moreover, encoding OR logic elements within RNA regions that are translated has required long open-reading frames with high secondary structure placed immediately upstream of the output gene sequence (Extended Data Fig. 1b). These regions can impede ribosome processivity, causing wide variations in the expression levels of the output gene depending on the input RNA^11^, and lead to extended N-terminal peptide extensions with unpredictable effects on output protein folding (Extended Data Fig. 1b,c). Addressing these limitations requires alternative means of initiating RNA–RNA interactions to reduce sequence constraints and improved strategies for encoding molecular logic that minimize their impact on output gene sequence.

**Fig. 1:**
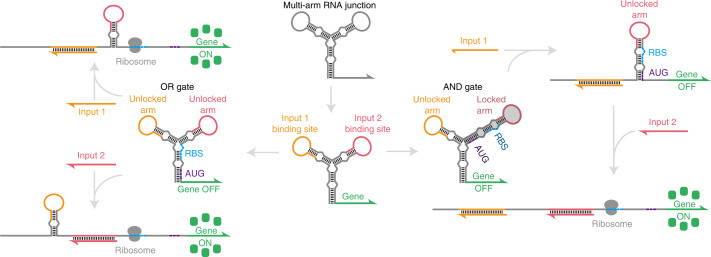
Design strategies for implementing sequence-independent RNA-based logic using multi-arm junctions. The three-arm junction in the schematic provides two sensor arms for binding to complementary input RNAs and is positioned upstream of the regulated gene. Logic operations are encoded within the multi-arm junction structure by controlling the length and number of the stem-loop arms. Unlocked stem-loop arms enable direct binding of input RNAs and are used for OR logic. Locked arms are not available for binding to an input RNA until they are unlocked through binding of another input RNA species and are used for AND logic.

Herein we describe a strategy for implementing molecular logic that exploits multi-arm junction RNA structures to regulate gene expression while eliminating input RNA sequence constraints and reducing sequence interference with the output gene for OR logic. These ribocomputing systems make use of loop-initiated RNA activator (LIRA) motifs that bind to input RNAs through extended loop domains and expose downstream functional domains for subsequent reactions. We show that LIRAs can be used as riboregulators to activate gene expression with high dynamic range and orthogonality in *Escherichia coli* cells without imposing any sequence constraints on input RNAs and the output gene. Using these validated motifs, we generate logic gate RNAs that encode multi-input molecular logic by folding up single strands of RNA into multi-arm junctions actuated by independent LIRA modules (Fig. 1). The resulting gate RNA structures unfold in a prescribed manner as they interact with cognate input RNAs to activate gene expression when AND and OR logic expressions are satisfied. We use the multi-arm junctions to implement three-input AND and OR operations in *E. coli* using completely sequence-independent input RNAs. Porting these systems to paper-based cell-free transcription–translation reactions, we find that LIRAs can be designed to detect viral RNA sequences and coupled with isothermal amplification reactions to detect the dengue virus, norovirus and yellow fever virus. Finally, we harness the capacity of multi-arm RNA junctions to detect sequence-independent inputs to implement paper-based molecular logic assays that use OR logic to tolerate sequence differences between human immunodeficiency virus (HIV) subtypes and AND logic to accurately identify severe acute respiratory syndrome coronavirus-2 (SARS-CoV-2) in clinical saliva samples by targeting two regions of the virus at the same time.

## Results

### Multi-arm junctions for controlling gene expression

Our general strategy for regulating gene expression using multi-arm junctions is illustrated in Fig. 1. A multi-arm structure is placed at the 5’ end of an mRNA and is followed by the coding sequence of the regulated gene. This configuration establishes strong secondary structures in the mRNA that conceal the ribosome binding site (RBS) and start codon (AUG) necessary to initiate translation. As a result, translation is repressed unless complementary input RNAs bind and unwind the structure. The stem-loop arms of the structure act as sensors that provide binding sites for each of the input RNAs. The form of molecular logic evaluated by the multi-arm assembly is programmed by controlling the length and number of the sensor arms. OR logic is implemented using short sensor arms that remain ‘unlocked’ and available for input RNA binding. Hybridization of either input RNA unwinds the cognate sensor arm through to the base stem to activate translation. To implement AND logic, all but one sensor arm is lengthened to establish a ‘locked’ configuration that prevents binding by the corresponding input RNA. Binding of successive inputs unlocks additional sensor arms for input binding and ultimately allows the RNA strand to be fully unwound, activating translation of the downstream gene. Compared to other approaches for implementing logic-gated gene expression with RNAs, this strategy abolishes the need for any sequence correlations between input RNAs for AND operations. Moreover, it does not require extended N-terminal residues to be added to the output protein for OR operations, which can interfere with protein folding. Implementing this strategy, however, first required that we develop loop-based RNA–RNA interactions that functioned reliably in vivo to enable unwinding of the junction structure through binding to the sensor arms.

### Loop-initiated translational activation

**Fig. 2:**
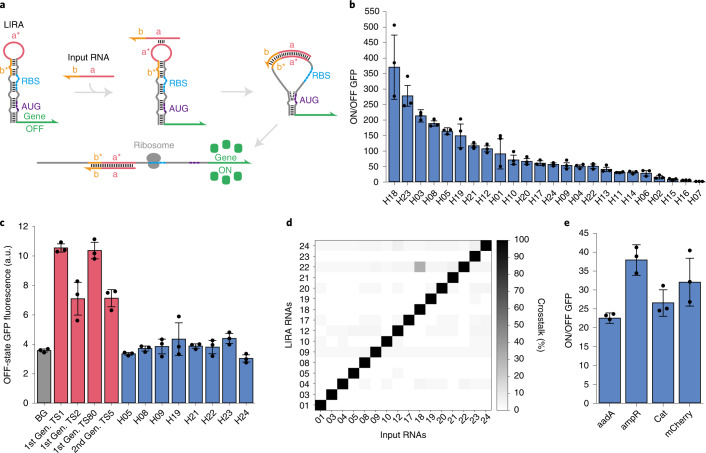
Design and in vivo validation of LIRAs. **a**, Schematic of the LIRA design and its interaction mechanism with the input RNA. Binding between the input RNA and the LIRA loop domain triggers release of the RBS and the start codon (AUG) to activate translation. **b**, ON/OFF fluorescence ratios of a library of 24 different LIRAs. **c**, Leakage comparison of LIRAs and four toehold switches measuring GFP fluorescence in the absence of the input RNA. **d**, Crosstalk evaluation for 16 selected LIRA devices. **e**, Detection of full-length mRNAs using LIRAs. Measurements were taken 3 h after induction with IPTG, *n* = 3 biological replicates, bars represent the arithmetic mean ± s.d. for **b** and **e** and the geometric mean ± s.d. for **c**.

**Extended Data Fig. 2:**
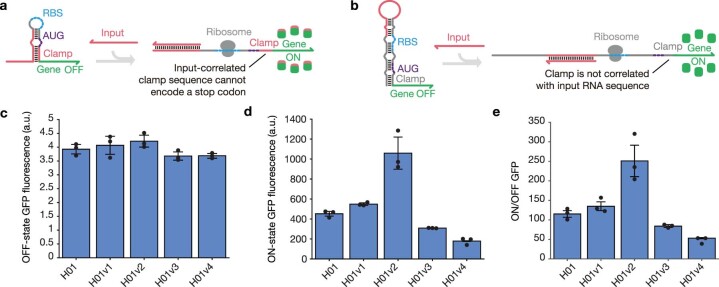
Comparison of sequence constraints for toehold switches and LIRAs. **a**, The toehold switch contains a 9- to 12-nt stem sequence immediately after the start codon that is defined by the sequence of the input RNA. This clamp region cannot encode an in-frame stop codon, which in turn imposes sequence constraints on the input RNA sequence. The clamp also adds three to four N-terminal residues to the output protein. **b**, LIRAs employ a 6-bp clamp domain downstream of the start codon to achieve low translational leakage with high ON-state signals. This clamp domain is not correlated with the input RNA sequence but does incorporate two additional residues into the N-terminus of the output protein. **c**, Experimental study of the effect of different clamp sequences on OFF-state GFP fluorescence of four LIRA H01 variants with different clamp sequences. OFF-state expression is unaffected by clamp sequence. **d**, Effect of different clamp sequences on the ON-state GFP fluorescence for LIRA H01 variants. **e**, ON/OFF fluorescence of LIRA H01 clamp variants. Significant variations in ON-state GFP and ON/OFF levels do occur, but all systems show ON/OFF levels greater than 50-fold. These results demonstrate that LIRAs can accommodate changes in clamp sequence and output protein N-terminal residues, while displaying good performance. n = 3 biological replicates, bars represent the geometric mean ± s.d. for **c** and **d** and the arithmetic mean ± s.d. for **e**.

We thus developed a set of riboregulators designed to be integrated into the stem-loop regions of multi-arm junctions. While many recent high-performance riboregulators, such as toehold switches^7^, have relied on single-stranded toehold domains to initiate reactions^7,36,38^, we hypothesized that long loop domains could be utilized to provide similar performance. Such long loops would provide a strong thermodynamic driving force to initiate RNA–RNA interactions and provide an input RNA binding site that is sufficiently labile and unconstrained to offer good reaction kinetics. Figure 2a shows the resulting loop-initiated RNA activators (LIRAs) that feature hairpins with extended loop domains regulating the expression of a downstream output gene. In the absence of the input RNA, translation by the LIRA is strongly repressed by sequestering both the RBS and the start codon of the output gene within the RNA duplex of the hairpin structure. A long loop domain a* of 21 nt is incorporated into the hairpin structure to promote the initial interaction between the LIRA and the activating RNA. After binding to the loop through the complementary a* sequence, the input RNA is designed to bind into the b* domain at the top of the hairpin stem, disrupting the existing base pairs and driving apart those located lower in the hairpin stem. Importantly, this effect enables the release of base pairs, in this case the RBS and the start codon that are completely unrelated to the sequence of the cognate input RNA. Thus, LIRAs can accommodate input RNAs without imposing any sequence constraints, and they can regulate a variety of different proteins without requiring modifications to the N-terminal sequence. In comparison, the design of toehold-switch riboregulators causes three to four N-terminal residues in the output protein to be defined by the sequence of the input RNA (Extended Data Fig. 2a), which prevents recognition of input RNAs that would generate stop codons upstream of the output gene^7^. To enable efficient expression and testing in vivo, four bulges were incorporated into the LIRA hairpin structure to reduce the likelihood of premature rho-independent transcriptional termination and to increase the thermodynamics driving the input–LIRA reaction. A 6 bp clamp domain was also added immediately after the start codon in the LIRA stem to reduce the likelihood of translational leakage (Fig. 2a and Extended Data Fig. 2b,c).

A library of 24 different LIRA sequences were designed de novo using the NUPACK software package^39^ (Methods) and plasmids were constructed to express the input and LIRA transcripts using T7 RNA polymerase in *E. coli* BL21 Star DE3 cells (Supplementary Tables 1 and 2 for primer and LIRA sequence information). These experiments employed green fluorescent protein (GFP) as the reporter protein and measured fluorescence from the cells using flow cytometry. ON/OFF ratios for the LIRAs were determined by measuring the ON-state GFP expression in the presence of the cognate input and dividing it by the GFP expression measured in the OFF state where a non-cognate input was expressed in the cell (Fig. 2b, LIRA ON and OFF values are shown in Supplementary Fig. 1). We found that 16 out of 24 of the LIRAs provided ON/OFF ratios over 50-fold, with the highest one yielding an ON/OFF ratio of ~350-fold. Additional tests using input RNAs complementary to different regions of the LIRA hairpin and different loop domain lengths revealed that the base LIRA design with a loop of 21 nt and a 31 nt input RNA provides the best overall performance (Supplementary Figs. 2 and 3, and Tables 3 and 4 for sequence information).

**Extended Data Fig. 3:**
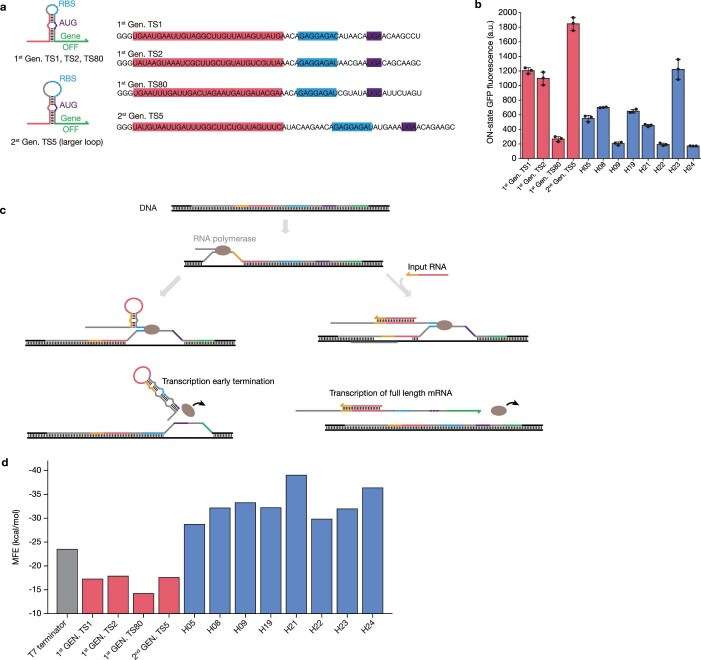
Transcriptional regulation of LIRAs and comparison to toehold switches. **a**, Structure and sequences of selected toehold switches used for testing translational leakage in Fig. 2c. **b**, ON-state GFP fluorescence of toehold switches and LIRAs tested for translational leakage in Fig. 2c. n = 3 biological replicates, bars represent the geometric mean ± s.d. **c**, Schematic of the putative transcriptional regulation mechanism of LIRAs. The strong hairpin structure of the LIRA can cause early transcriptional termination for some transcripts. In the presence of the input RNA, binding of the input prevents formation of the hairpin structure and promotes transcription of the full-length mRNA. **d**, Minimum free energy (MFE) of the T7 terminator and the hairpin structures of the toehold Switches and LIRAs from Fig. 2c. Toehold switches have a weaker secondary structure than the T7 terminator, which makes them unlikely candidates for transcriptional termination. LIRAs possess a longer hairpin structure and have a stronger secondary structure than the T7 terminator. The stronger hairpin structure is expected to encourage transcriptional termination, albeit at a lower rate than natural terminators that feature 3’ U-tracts and short loop domains to promote faster hairpin formation.

We observed that multiple LIRAs provided very low translational leakage in the absence of the input RNA. Figure 2c shows the OFF-state GFP fluorescence of eight different LIRAs with ON/OFF ratios greater than 50 compared with the autofluorescence of cells lacking GFP and a set of previously reported toehold-switch riboregulators with wide dynamic range^7^ (Extended Data Fig. 3a). We found that the toehold switches in the OFF state yielded 2- to 3-fold higher fluorescence than the cells lacking GFP plasmids. In contrast, all eight of the LIRAs examined provided fluorescence leakage that was statistically indistinguishable from the background cellular fluorescence, with *P* > 0.067 for all the LIRA devices in the plot compared with cell autofluorescence. Comparison of ON-state signals showed that three out of the four toehold switches provided higher signal output than the LIRAs (Extended Data Fig. 3b). To explain the very low OFF-state signals, we hypothesized that low-leakage LIRAs could be making use of a combined translational and transcriptional regulation mechanism to yield virtually undetectable leakage (Extended Data Fig. 3c), assisted by the strong secondary structure of the LIRA hairpin (see Extended Data Fig. 3d for comparison of minimum free energies for LIRA and toehold-switch hairpins). To verify this hypothesis, we performed reverse transcription quantitative polymerase chain reaction (RT-qPCR) experiments to measure the concentration of LIRA RNAs and cognate and non-cognate input RNAs expressed from cells in the ON and OFF states, respectively (Supplementary Fig. 4). We found that expression of the LIRA transcript with a non-cognate input was only 25% of that measured for the transcript with a cognate input, confirming that part of the LIRA regulation is due to transcriptional control. We also studied a set of four LIRA variants that contained different sequences in the stem below the start codon (see Supplementary Table 5 for sequence information), which in turn modify the N-terminal residues in the output protein. We found that these clamp sequence changes did not impact the OFF-state signal of the LIRAs (Extended Data Fig. 2c), but they did cause variations in ON-state expression levels ranging from 40% to 230% of the parent LIRA (Extended Data Fig. 2d). Despite these variations, all of the devices with clamp modifications displayed ON/OFF ratios of at least 50-fold (Extended Data Fig. 2e), indicating that changes in clamp sequence and the N-terminal residues are well tolerated by the riboregulators.

Foreshadowing their use in multi-arm junctions, we evaluated LIRA orthogonality by measuring the crosstalk observed between the 16 devices providing the widest dynamic range. A 16 × 16 matrix of pairwise LIRA–input RNA interactions was measured by transforming cells with different combinations of plasmids. Figure 2d shows the measured crosstalk between the devices. Cognate interactions along the diagonal are normalized to 1 for the riboregulators in their ON states, while off-diagonal, non-cognate interactions reflect the percent activation with respect to the ON state. We found that crosstalk from the non-cognate inputs was very low, less than 4% in nearly all cases, with a single strong off-target interaction observed between LIRA 22 and input 18 showing 5.6% crosstalk. Thus, the LIRAs provided a set of 15 orthogonal devices for regulation of gene expression in vivo.

On the basis of their low crosstalk and lack of sequence constraints, we also investigated whether LIRAs could be designed to detect mRNAs within the cell. A set of LIRAs targeting regions of low secondary structure in the mRNAs for *mCherry* and the antibiotic resistance genes *aadA*, *ampR* and *cat*, conferring resistance to spectinomycin, ampicillin and chloramphenicol, respectively, was investigated (see Supplementary Table 6 for sequence information). All LIRAs were based on a high-performance design identified during library screening and were generated simply by replacing the original target-binding site with the reverse complement of the mRNA target site. We found that all four mRNAs could be readily detected using the LIRAs and provided ON/OFF GFP levels ranging from 22- to 38-fold (Fig. 2e).

### Multi-arm RNA junctions for in vivo molecular logic

**Fig. 3:**
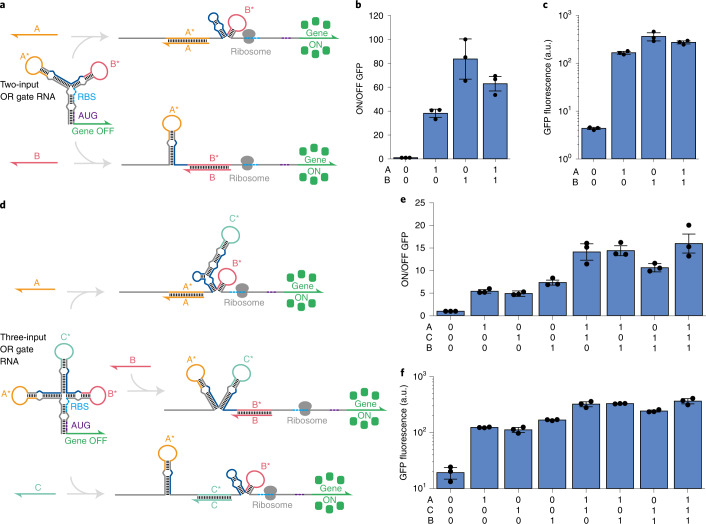
Multi-arm RNA junction molecular OR logic in *E. coli*. **a**, Schematic of a two-input OR logic gate RNA and its interaction mechanism with the input RNAs. **b**, ON/OFF fluorescence ratios of each input combination. **c**, GFP fluorescence of each input combination. **d**, Schematic of a three-input OR logic design and its interaction mechanism with the input RNAs. **e**, ON/OFF fluorescence ratios of each input combination. **f**, GFP fluorescence of each input combination. All *P* values from two-tailed Student’s *t*-tests between each TRUE state and FALSE state are less than 0.0174. Measurements were taken after 4 h of IPTG induction, *n* = 3 biological replicates, bars represent the arithmetic mean ± s.d. for **b** and **e** and the geometric mean ± s.d. for **c** and **f**.

Having developed a set of orthogonal LIRAs lacking sequence constraints, we next integrated them as sensor modules into the multi-arm RNA junction structures for computing intracellular OR and AND logic expressions. The sensor arms of the resulting logic gate RNA are each capped by different LIRA modules and designed to direct the unfolding of the structure as input RNAs bind to the gate RNA. Two-input OR logic devices were constructed upstream of a GFP reporter using a three-arm junction containing a pair of LIRA sensor arms (Fig. 3a). The base arm contains the RBS and start codon signals topped by the LIRA arms to provide binding sites A* and B* for interaction with the complementary input RNAs A and B. Binding of either input RNA disrupts the cognate stem-loop structure and further draws apart the base arm to reveal the RBS and start codon for translation initiation. To increase translational output for this input and reduce the likelihood of transcriptional regulation, we also incorporated a hairpin reconfiguration domain (indicated in dark blue in Fig. 3a) that generated an additional stem-loop upon binding of input A to the gate RNA. This newly formed stem disrupted the bottom grey portion of the input B LIRA module, providing a single-stranded region upstream of the RBS and greater space to better accommodate the ribosomal footprint. During transcription, the hairpin domain can also help delay formation of the strong LIRA stem-loop structures to discourage transcriptional termination.

We tested the two-input OR device by transcribing the input and gate RNAs off separate high- and medium-copy plasmids, respectively, in *E. coli* (see Supplementary Table 7 for sequence information). Using flow cytometry, we found that GFP expression increased by 38- to 84-fold when any combination of the two-input RNAs was expressed (Fig. 3b,c). We also constructed a three-input OR gate RNA using three orthogonal LIRA modules (Fig. 3d). This four-arm junction system contained a base arm with the RBS and the start codon and inserted the LIRA stem loop for input C between modules for inputs A and B. Similar to the two-input device, the two left LIRA stem loops also contained hairpin reconfiguration domains to enable increased translation upon binding of inputs A and C. This circuit also performed as expected in vivo, with low expression for the null-input logical FALSE case and 6- to 19-fold increases in expression when the input RNAs were expressed in any combination (Fig. 3e,f).

**Fig. 4:**
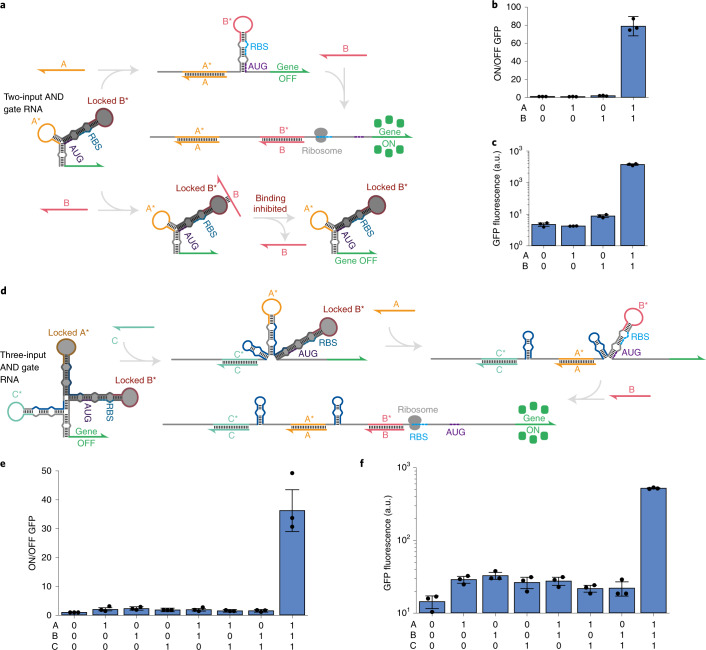
Multi-arm RNA junction molecular AND logic in *E. coli*. **a**, Schematic of the two-input AND logic design and its interaction mechanism with the input RNAs. **b**, ON/OFF fluorescence ratios for each input combination. **c**, GFP fluorescence for each input combination. **d**, Schematic of the three-input AND logic design and its interaction mechanism with the input RNAs. **e**, ON/OFF fluorescence ratios for each input combination. **f**, GFP fluorescence for each input combination. All *P* values from two-tailed Student’s *t*-tests between each FALSE state and TRUE state are less than 0.0251. Measurements were taken after 4 h of IPTG induction, *n* = 3 biological replicates, bars represent the arithmetic mean ± s.d. for **b** and **e** and the geometric mean ± s.d. for **c** and **f**.

Multi-arm junctions for AND logic employ sensor arms of different strengths to implement locked and unlocked LIRA sites (Fig. 4a). The gate RNA contains a base arm topped by a weak, unlocked sensor arm for LIRA module A* and a strong, locked sensor arm for LIRA module B*. The locked arm also conceals the RBS and the start codon translation initiation signals within an RNA duplex. For the logical FALSE case when only input B is expressed, the locked stem-loop structure and the base stem are designed to be too thermodynamically stable to be disrupted by input RNA B, preventing system activation. However, if input A interacts with the gate RNA first, its binding energy is sufficiently strong to disrupt both the left stem loop and the base stem of the gate RNA. Unwinding the base stem in turn unlocks the LIRA B* module, making it sufficiently weak to interact with input B. Thus, when input B is also expressed, the B* module is completely disrupted and the RBS and start codon are exposed for translation of the GFP reporter gene. Unlike the LIRA OR gates, use of locked sensor arms for LIRA AND gates does add multiple N-terminal residues to the output protein (Fig. 4a and Extended Data Fig. 1c).

We tested the two-input AND device in *E. coli* using different combinations of input RNAs (see Supplementary Table 7 for sequence information). We found that only strong GFP reporter expression was observed for the logical TRUE case with both inputs expressed. GFP expression increased by 79-fold for the TRUE case compared with the case with neither input transcribed (Fig. 4b,c). In addition, we found that translational leakage in the presence of input RNA B was low, 43-fold lower than the TRUE state, indicating that the extended stem-loop structure effectively blocked access of the transcript to the gate RNA. We also extended the AND ribocomputing strategy to three inputs using the four-arm junction structure shown in Fig. 4d. This device incorporated the binding site for input C to lock modules A* and B* and prevent them from interacting with their corresponding input RNAs without expression of input C. To increase translational output and encourage stem-loop disruption, hairpin reconfiguration domains were added to the arms for inputs A and C. This device also functioned properly in *E. coli*, providing a 36-fold increase in GFP expression in the logical TRUE case with all three inputs expressed compared with the null-input case (Fig. 4e,f). Leakage in all logical FALSE conditions was low, with the TRUE state providing at least 16-fold higher GFP output in all cases.

### Validation of LIRAs in paper-based diagnostics

**Fig. 5:**
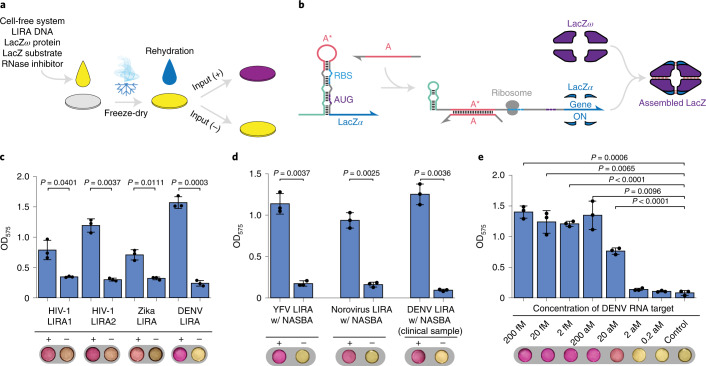
Pathogen-detecting LIRAs in paper-based cell-free assays. **a**, Schematic of paper-based diagnostic assays where cell-free transcription–translation reactions are freeze dried on paper discs to stabilize them at room temperature. Paper-based systems are reactivated by adding water with the RNA analyte of interest. **b**, LIRA design used for detection of viral RNAs in paper-based reactions. The optimized LIRA contains a 5’ hairpin reconfiguration domain that forms after input RNA binding to assist with activating the LIRA and increasing output gene expression. **c**, Detection of synthetic RNA targets for HIV, the Zika virus and the dengue virus (DENV) in 80 min paper-based reactions. The purple colour change of the discs is measured by the optical density at 575 nm (OD_575_) and indicates that the input RNA has been detected. **d**, Detection of viral RNA targets at initial concentrations of 200 aM after coupling with NASBA isothermal amplification and running cell-free reactions for 80 min for YFV and 90 min for norovirus. Clinical serum samples positive and negative for DENV were amplified by NASBA and detected after 90 min in paper-based reactions containing a DENV-specific LIRA. **e**, Detection limit test of DENV at different starting concentrations of synthetic input RNAs, demonstrating a limit of detection of 20 aM after 90 min paper-based cell-free reactions. Precise *P* values from two-tailed Student’s *t*-test are indicated. *n* = 3 technical replicates, bars represent the arithmetic mean ± s.d. for **c**, **d** and **e**.

The sensing and logic capabilities of LIRAs and multi-arm junction RNA structures also make them promising devices for use in paper-based cell-free systems, where they can be used as diagnostics without the need for expensive equipment and provide results that can be detected by the naked eye^14–17^. Since RNA–RNA interactions differ in cell-free reactions compared with the cytoplasmic environment, we first tested LIRAs by using them as riboregulators in paper-based reactions (see Supplementary Table 8 for sequence information). These reactions employed freeze-dried cell-free transcription–translation reactions along with LIRA plasmids, the lacZ *ω* subunit and the lacZ colourimetric substrate CPRG (chlorophenol-red-*β*-d-galactopyranoside) deposited onto 2-mm-diameter paper discs (Fig. 5a). At the time of use, the paper discs were rehydrated with solutions containing RNAs for detection by the embedded LIRA riboregulators. We first tested the paper-based reactions with LIRAs that showed wide dynamic range during in vivo experiments. However, in the cell-free reactions, they were unable to be turned on by their cognate input RNAs. To increase translational output and encourage stem-loop disruption, a hairpin reconfiguration domain was added to the 5’ end of each LIRA sensor (Fig. 5b). Applying synthetic viral RNA targets to a final concentration of 5 µM, we found that the updated LIRA pathogen sensors provided strong increases in absorbance at 575 nm wavelength as the yellow-to-purple CPRG cleavage reaction was carried out by lacZ (Fig. 5c). Reactions with the pathogen RNAs turned to the expected pink or purple colour as the reactions proceeded, while those without the pathogen RNAs remained yellow to yellow-pink in colour depending on the sensor (Fig. 5c, bottom).

To enable detection of RNAs at the concentrations typically present in clinical samples, we used nucleic acid sequence-based amplification (NASBA) to amplify low-concentration pathogen RNAs before use in the paper-based assays. In NASBA, a combined reaction featuring reverse transcription, T7 RNA polymerase, RNase H and DNA primers that incorporate the T7 promoter sequence is used to generate multiple RNA copies from a starting RNA template. Synthetic RNA targets from norovirus and yellow fever virus (YFV) were supplied to NASBA reactions at an initial concentration of 200 aM and amplified over 2 h at 41 °C. We found that both pathogen RNAs could be detected in the colourimetric paper-based reactions following NASBA (Fig. 5d).

In addition, we applied the assay to clinical serum samples positive and negative for the dengue virus. The serum samples were first diluted by 10-fold into water and heated to 95 °C for 2 min to release the viral genome from the capsid. The RNA was then amplified using NASBA and applied to the paper-based LIRA sensors. We found that LIRAs could unambiguously identify the clinical dengue sample through the resulting purple colour. To determine the detection limit of the dengue assay, we carried out a series of NASBA/LIRA reactions with synthetic dengue target RNA concentrations ranging from 200 fM down to 0.2 aM. We found that the dengue transcript could be detected down to concentrations as low as 20 aM in the NASBA reaction, which corresponds to 12 RNA copies per µl of reaction (Fig. 5e).

### Paper-based diagnostic with embedded molecular logic

Diagnostic devices that combine visible readouts with the ability to perform information processing on biomolecular inputs have the potential to improve assay capabilities by expanding the number of pathogens a single test can detect, reducing false positives, and lowering assay complexity and cost. To demonstrate the potential of such logic-enabled paper-based diagnostic devices, we carried out proof-of-concept studies exploiting the logic capabilities of multi-arm junction molecular logic for HIV and SARS-CoV-2 detection. HIV continues to be a major global health threat with HIV-1 group M being the predominant cause of infections worldwide^40,41^. Within group M, there are nine different subtypes with genetic distances of 25% to 35% and prevalences that vary depending on the geographic region. HIV-1 subtype C causes >50% of infections worldwide and circulates mostly in India and regions of Africa, while HIV-1 subtype B predominates in Europe and the Americas^42^. We thus aimed to develop a logic system capable of detecting both HIV-1 subtype B and C using a single OR operation, which could be deployed in an area such as Southern Brazil where both subtypes are common^43^.

**Fig. 6:**
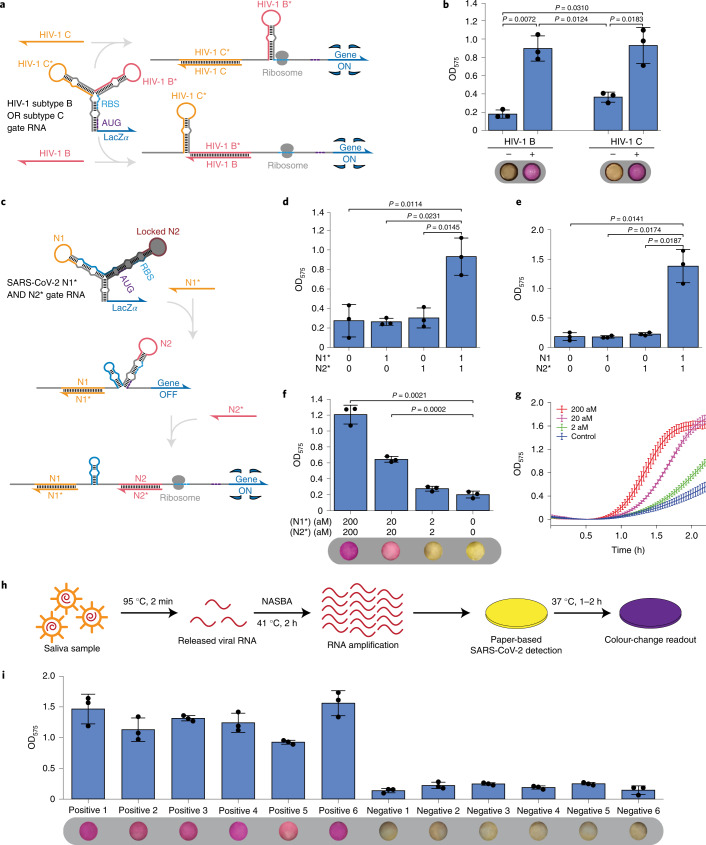
Logic-enabled paper-based tests using multi-arm junction gate RNAs for identification of HIV-1 group M subtypes and SARS-CoV-2. **a**, Schematic of the three-arm junction gate RNA used for simultaneous detection of HIV-1 B and C subtypes via two-input OR logic. **b**, Detection of HIV-1 B and C subtypes in paper-based reactions. Photographs were taken after 90 min of the cell-free reaction. **c**, Schematic of the three-arm junction gate RNA used for SARS-CoV-2 detection via two-input AND logic. **d**, OD_575_ for an AND gate RNA N1*N2* detecting synthetic input fragments N1* and N2* from the SARS-CoV-2 genome. **e**, OD_575_ for an AND gate RNA N2*N1 detecting synthetic input fragments N2* and N1 from the SARS-CoV-2 genome. **f**, Validation of AND gate RNA N1*N2* with heat-inactivated SARS-CoV-2 virions amplified via isothermal NASBA reactions. Inactivated viruses were diluted with water. Photos were taken after 90 min reactions. **g**, Time-course curves for the reactions tested in **f**. **h**, Schematic of the process for measuring clinical saliva samples starting from heat-driven RNA extraction through to the colourimetric paper-based cell-free assay. **i**, AND gate RNA N1*N2* tested with SARS-CoV-2 positive and negative saliva samples with NASBA products after 2 h reactions, *P* values between each pair of positive and negative samples are all less than 0.0145. Precise *P* values from two-tailed Student’s *t*-tests are indicated. *n* = 3 technical replicates, bars represent the arithmetic mean ± s.d. for **b**, **d**, **e**, **f** and **i**, curves represent the arithmetic mean ± s.d. for **g**.

To implement the system, we first identified conserved regions in the genomes of HIV-1 subtypes B and C to use as circuit input RNAs. Complementary sequences for these inputs were then incorporated into a two-input OR three-arm junction gate RNA (Fig. 6a, see Supplementary Table 9 for sequence information). To ensure the system functioned properly in paper-based cell-free reactions, the binding site for the input RNAs was extended so that it included both the loop domain and the entire stem of the LIRA module. The gate RNA was transcribed in the cell-free reactions and supplied with the HIV-1 subtypes B and C input RNAs. For both inputs, output of the lacZ *α* subunit was produced as evidenced by increased production of the purple cleavage product in the paper-based reactions (Fig. 6b). Reactions lacking either input RNA remained yellow.

We next made use of AND logic operations to implement RNA devices for SARS-CoV-2 detection. SARS-CoV-2, which was first reported in 2019 in Wuhan, China, has now become a global pandemic with over 100 million reported cases and over 3 million deaths worldwide according to data from the Johns Hopkins Coronavirus Resource Center. SARS-CoV-2 can be transmissible even before any symptoms have developed^44,45^ and studies have shown that many patients who test positive for the virus do not show any symptoms^46^. These factors have allowed the pandemic to take hold and emphasize the importance of developing diagnostic assays that can be widely deployed to detect SARS-CoV-2, even in carriers who do not have any signs of illness.

Following the US Centers for Disease Control and Prevention (CDC) recommendations^47^, SARS-CoV-2 infections are often identified by amplification of two selected regions of the virus nucleocapsid (N) gene, 2019-nCoV_N1 and 2019-nCoV_N2. RT-qPCR is the most common method of detection of SARS-CoV-2 given its excellent specificity and sensitivity. However, it requires well-trained personnel and expensive equipment, which makes virus detection more challenging in rural areas with limited medical resources and requires additional time to ship samples to centralized facilities. Previous paper-based cell-free assays have been limited to detecting only a single pathogen target sequence at a time, and parallel assays that detect target RNAs in separate reactions can suffer as a result of differences in riboregulator activation speeds and lead to increased assay cost.

To overcome these issues, we combined AND logic multi-arm junctions with isothermal amplification reactions to simultaneously detect two different SARS-CoV-2 N gene sequences using a single paper-based readout reaction. The resulting two-input AND gate RNAs contained a hairpin reconfiguration domain to encourage binding between the gate RNA and the input viral RNAs (Fig. 6c, see Supplementary Table 9 for sequence information). We first evaluated several devices using synthetic targets and identified two with the best performance. Gate RNA N1*N2* recognizes the antisense sequences in regions N1 and N2 of the SARS-CoV-2 N gene, with the left and right sensor arms targeting N1* and N2*, respectively (Fig. 6d). Similarly, gate N2*N1 targets the antisense sequence of the N2 region with the left sensor arm and the sense sequence of the N1 region with the right sensor arm (Fig. 6e). Both devices show clear colour changes in the presence of the two-input RNAs, but did not activate when one or both inputs were absent, thus carrying out AND logic (Fig. 6d,e).

**Extended Data Fig. 4:**
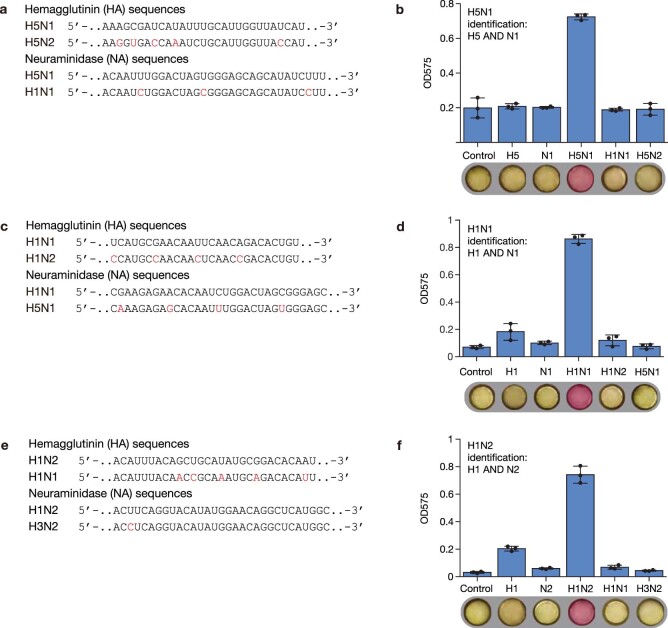
Multi-arm junction gate RNAs for identification of influenza A subtypes. **a**, Sequences of H5N1 and related subtypes. **b**, Differentiation of H5N1 from related subtypes in 2-h paper-based reactions. **c**, Sequences of H1N1 and related subtypes. **d**, Differentiation of H1N1 from related subtypes in 2-hour paper-based reactions. **e**, Sequences of H1N2 and related subtypes. **f**, Differentiation of H1N2 from related subtypes in 80-minute paper-based reactions. n = 3 technical replicates, bars represent the arithmetic mean ± s.d. for **b**, **d** and **f**.

We then designed specific NASBA primer pairs for each of the two devices to amplify the input RNAs from the SARS-CoV-2 genome. NASBA reactions were performed using heat-inactivated SARS-CoV-2 virus particles at different concentrations. We found that gate N1*N2* performed better than gate N2*N1 and enabled detection of SARS-CoV-2 down to concentrations of 20 aM in the NASBA reactions when viewed by the naked eye (Fig. 6f,g), a concentration that is within the range necessary for detecting the virus in clinical samples^48^. Using a plate reader, SARS-CoV-2 down to a concentration of 2 aM in the NASBA reaction could be distinguished. We then tested six positive saliva samples from SARS-CoV-2 patients together with six negative ones. Figure 6h illustrates the process from sample treatment to paper-based reactions. Diluted saliva samples were subjected to a brief 95 °C heating step for 2 min to release the viral RNA and then added to NASBA reactions for amplification of each input RNA. The resulting amplicons were then applied to paper-based cell-free reactions for testing with the SARS-CoV-2 AND gate N1*N2* RNA. As shown in Fig. 6i, the gate RNA detected the six positive samples, generating a clearly visible purple colour, while the six negative samples remained yellow in colour. A similar strategy was also applied to differentiate influenza A subtypes and distinguished H1N1, H5N1 and H1N2 from closely related virus subtypes (Extended Data Fig. 4, see Supplementary Table 10 for sequence information).

## Discussion

We have implemented a strategy for encoding molecular logic operations in multi-arm RNA junctions for regulation at the translational level. These systems make use of loop-initiated RNA–RNA interactions via LIRA modules to detect input RNAs and direct the programmed unfolding of the multi-arm RNA structures to report on computation results. We have found that LIRAs on their own can operate as riboregulators with wide dynamic range, good orthogonality and low translational leakage using such loop interactions. Moreover, they completely decouple the sequence of their cognate input RNA from the sequence of the output module that they expose, thereby avoiding some of the limitations of toehold-based riboregulators. By incorporating LIRA modules into the multi-arm junctions, we implemented three-input OR and three-input AND operations in living *E. coli* cells. We also applied these systems in paper-based cell-free assays for detection of viruses, including the dengue virus and SARS-CoV-2 from clinical samples. Using multi-arm junctions in paper-based reactions, we produced colourimetric assays that harness OR logic to activate in response to two different subtypes of HIV-1 and AND logic to target two regions of the nucleocapsid gene of SARS-CoV-2 at the same time. Application of the system to a set of positive and negative saliva samples demonstrated accurate identification of SARS-CoV-2 using a two-input multi-arm junction gate RNA.

Our results show that loop-initiated interactions can be very effective at driving RNA–RNA interactions in vivo and in paper-based cell-free reactions. However, effective interactions require loop domains that are sufficiently long (≥15 nt, see Supplementary Fig. 2) to provide effective binding sites and sufficient binding free energy to promote capture of the input RNA. Invasion of the input RNA into the regulator stem region further promotes the interaction and helps drive apart the remaining base pairs in the stem. In comparison with ribocomputing devices based on toehold switches, our results indicate that LIRA-based molecular logic systems generally provide lower ON/OFF ratios, probably because loop-initiated interactions are not quite as effective as toehold-initiated ones. Despite this disadvantage, we do find that LIRA-based systems offer several key benefits over toehold-switch-based circuitry^11^. The use of multi-arm junctions for LIRA OR gate RNAs does not require translation through downstream hairpin structures and alleviates the need for long N-terminal peptides to be added to the output protein. These design features for toehold-mediated OR gates result in N-terminal peptides that increase in size by about 24 residues for each additional input detected (Extended Data Fig. 1c). This condition has led to the fusion of N-terminal peptides up to 123 residues long to the reporter protein^11^, which corresponds to nearly half the length of GFP. Moreover, these OR gates can exhibit substantial variations in output protein expression as a function of the input RNA, yielding as much as a 14-fold difference in signal depending on the input used^11^. For the LIRA-based OR gates reported here, the peak variation in output expression observed is 3.2-fold (Fig. 3e). Since loop-initiated OR gate performance is dominated by effects at the RNA level, as opposed to less predictable factors such as ribosome processivity and N-terminal peptide folding and translation efficiency, we expect that future refinements in LIRA OR gate secondary structure and computational design will enable higher ON/OFF ratios to be achieved.

For LIRA-based AND gates, the use of loop-initiated interactions allows sets of completely unrelated input RNA sequences to be monitored, facilitating accurate detection of two SARS-CoV-2 targets in a single paper-based colourimetric reaction. Direct detection of two pathogen targets is not possible using previously reported cell-free toehold-switch assays as a result of input sequence complementarity requirements. Detection of two genomic sites simultaneously can also offer advantages compared with recently reported riboregulators with single-nucleotide specificity when targeting pathogens that are known to be mutating rapidly^17^. The multi-arm junction AND gate design does require some N-terminal residues to be added to the output protein. However, the length of the additional peptide is much smaller than that used for toehold-mediated OR gate RNAs. This peptide length increases at an expected rate of only about seven residues for each additional input (Extended Data Fig. 1c). Given the strengths and weaknesses of toehold- and loop-initiated interactions, we expect that future ribocomputing systems can make use of both of these strategies in the same gate RNA to achieve optimal performance by maximizing dynamic range, reducing expression variability and avoiding input sequence constraints.

CRISPR-based molecular diagnostics^49–51^ have also been applied for rapid detection of SARS-CoV-2^52,53^. These assays have demonstrated limits of detection of ~10 copies per µl in the sample^52^ compared with the 60 copies per µl (or 20 aM in the amplification reaction) reported in this work for visible detection. CRISPR-based visible readout reactions have relied on lateral flow strips and targeted a single viral site in each reaction^52,53^. Our strategy provides a simplified procedure by monitoring two SARS-CoV-2 amplicons in the same reaction. Since the paper-based riboregulator assays can be run in array formats^14^ and monitored directly using cameras without added light sources or filters, readout via colourimetric cell-free reactions could also enable parallel testing of larger numbers of samples than CRISPR-based assays.

We expect that the in vivo-validated loop-initiated motifs described here, which eliminate any correlation between the input and output sequence, will prove broadly useful for implementing a variety of other forms of RNA-based regulation, particularly those that can require strict sequence constraints, such as conditional guide RNAs^54–58^ and aptamer-based probes^18^. Moreover, the strategy for encoding molecular logic using multi-arm junction structures can also be applied to a variety of different forms of RNA output and provide the capacity to respond to multiple input species without sequence constraints. We anticipate that these capabilities will prove valuable for constructing intracellular systems that respond to endogenous RNAs to report and control cell state for biological circuits. In addition, they can be deployed in diagnostic assays to increase specificity and sensitivity, while reducing cost and test complexity to help respond to infectious disease outbreaks.

## Methods

### LIRA library design

LIRAs were designed computationally using the NUPACK software package^39^ and selected for experimental testing using procedures reported previously^7^. Briefly, a set of 337 candidate LIRA devices were generated by NUPACK and the top 60 of these riboregulators were selected on the basis of their ensemble defect levels. All candidate LIRAs shared the same secondary structures but differed in sequence outside of the conserved RBS, start codon and reporter gene regions. Pairwise interactions between all the LIRAs and input RNAs were then computed to determine the expected equilibrium concentration of the LIRA–input complexes formed. Using non-cognate LIRA–input complex formation probability as a crosstalk metric, a Monte Carlo selection algorithm was used to generate a library of 24 LIRAs expected to display the lowest expected overall crosstalk.

### Strains and growth conditions

The following *E. coli* strains were used in this study: BL21 Star DE3 (F^−^ *ompT hsdS*_*B*_ (r_B_^−^m_B_^−^) *gal dcm rne131* (DE3); Invitrogen), BL21 DE3 (*F*^*−*^ *ompT hsdSB* (r_B_^−^m_B_^−^) *gal dcm* (DE3); Invitrogen), MG1655Pro (F^−^ λ^−^ *ilvG- rfb-50 rph-1 Sp*^*R*^ *lacR tetR*), and DH5α (*endA1 recA1 gyrA96 thi-1 glnV44 relA1 hsdR17*(r_K_^−^m_K_^+^) λ^−^; Invitrogen). All strains were grown in Luria broth (LB) medium at 37 °C with appropriate antibiotics.

### Plasmid construction

Plasmids were constructed via PCR and Gibson assembly. Single-stranded DNAs for expressing LIRAs, gate RNAs and input RNAs were purchased from Integrated DNA Technologies and amplified into double-stranded DNA form via PCR. The amplified DNAs were then connected with plasmid backbones by 30 bp homology domains using Gibson assembly. All Gibson assembly products were transformed in the *E. coli* DH5α strain and sent out for sequence validation via Sanger sequencing. Backbones used for constructing the plasmids were amplified from the commercial vectors pET15b, pCOLADuet and pCDFDuet (EMD Millipore) via PCR followed by treatment with the restriction enzyme DpnI. The reporter protein for all plasmids is GFPmut3b with an ASV degradation tag unless otherwise noted.

### Flow cytometry measurements and analysis

Bacterial colonies transformed with combinations of LIRA or gate RNA and input RNA plasmids were inoculated in 1 ml of LB in triplicate with appropriate antibiotics and grown overnight at 37 °C with shaking. On the second day, 5 µl overnight-cultured medium was diluted 100-fold in 495 µl of fresh LB with 30 µg ml^−1^ kanamycin, 50 µg ml^−1^ ampicillin and 25 µg ml^−1^ spectinomycin. After 80 min of recovery, isopropyl β-D-1-thiogalactopyranoside (IPTG) was added into each well to a final concentration of 0.1 mM. Flow cytometry measurements were performed after 3, 4 and 5 h of induction.

Flow cytometry was performed using an S1000 cell analyser (Stratedigm) equipped with a high-throughput auto sampler (A600, Stratedigm). Before running measurements, cells were diluted ~10-fold into phosphate buffered saline (PBS) in 384-well plates. Forward scatter (FSC) was used for the trigger, and ~40,000 individual cells were recorded. Cell populations were gated according to their FSC and side scatter (SSC) distributions as described previously^7,33^ (see Supplementary Fig. 5 for representative gating data). The GFP fluorescence signal outputs of these gated cells were used for subsequent calculations. Error levels for the fluorescence measurements of ON-state and OFF-state cells were calculated from the s.d. of measurements from at least 3 biological replicates. The relative error levels for the ON/OFF fluorescence ratios were then determined by adding the relative errors of ON and OFF-state fluorescence in quadrature.

### Cell-free reactions

Cell-free transcription–translation systems (NEB, PURExpress) were prepared for freeze-drying according to the following recipe: cell-free solution A, 40%; cell-free solution B, 30%; RNase inhibitor (Roche, 03335402001, distributed by MilliporeSigma), 2%; chlorophenol-red-b-d-galactopyranoside (Roche, 10884308001, distributed by MilliporeSigma, 24 mg ml^−1^), 2.5%; with the remaining volume reserved for LIRA riboregulator or gate RNA plasmids, water and lacZ *α* peptide added to a final concentration of 2 µM. When testing LIRA riboregulators from a plasmid, the plasmid DNA was added to a final concentration of 30 ng µl^−1^ in the cell-free reaction mix. For the gate RNA devices test in the paper-based system, the final concentration of the plasmid was 15 ng µl^−1^.

Filter paper (Whatman, 1442-042) for depositing and freeze-drying of the cell-free system was first blocked with 5% bovine serum albumin overnight. The paper was washed three times in water for 5 to 10 min after overnight blocking, transferred on a hot plate at 50 °C for drying and then cut into 2-mm-diameter paper discs with a biopsy punch. The discs were then transferred into 200 µl PCR strips and 1.8 µl of the above cell-free reaction mix was applied to each of them. Liquid nitrogen was used to freeze the PCR strips containing these paper devices. The frozen paper discs were dried overnight in a lyophiliser. Plate reader tests were carried out on the freeze-dried paper discs 2–4 d later. The systems were stored in a nitrogen environment shielded from light and with the silica gel desiccation packages as described previously^14^. The paper discs remained active for at least a month under storage at room temperature.

### NASBA reactions

NASBA experiments were carried out using the following standard protocols: reaction buffer (Life Sciences, NECB-24; 33.5%), nucleotide mix (Life Sciences NECN-24; 16.5%), RNase inhibitor (Roche, 03335402001; 0.5%), 12.5 µM of each DNA primer (2%), nuclease-free water (2.5%) and RNA amplicon (20%) were assembled at 4 °C. After being incubated at 65 °C for 2 min and a 10 min incubation at 41 °C, 1.25 µl of enzyme mix (Life Sciences NEC-1-24; 25%) was added to the reaction. The reaction took place at 41 °C for 2 h and was then diluted 1:6 into water before applying 2 µl to the freeze-dried paper devices. For the dengue samples, de-identified clinical serum samples positive and negative for the virus were obtained at Salud Digna (Culiacan, Mexico) and provided as remnant biospecimens. The sample was first diluted 10-fold into water and then heated for 2 min at 95 °C for RNA release. The heat-extracted RNA was then added to the NASBA reaction. Heat-inactivated de-identified saliva samples positive and negative for SARS-CoV-2 were provided by the Arizona State University Biodesign Institute Clinical Testing Lab as remnant biospecimens. Heat inactivation was performed by incubating samples at 65 °C for 30 min. The saliva samples were diluted 1:1 into water and heated at 95 °C for 2 min before spiking in NASBA reactions. A 1 µl aliquot of each sample was transferred into 5 µl NASBA reactions. Each sample was amplified using separate NASBA reactions with the corresponding primer pairs designed for each input RNA. After a 2 h reaction, 1 µl of each pair of NASBA products was combined and diluted with 5 µl water before adding 2 µl to the paper-based cell-free reaction.

### RT-qPCR reactions

Primers were designed for both the *GFP* gene and 16S rRNA, which was used as the internal control (see Supplementary Table 11 for primer sequence information). Colonies with bacteria transformed with LIRA plasmids, toehold-switch plasmids, and cognate or non-cognate input plasmids were inoculated into 6 ml LB in triplicate with appropriate antibiotics. Total RNA was extracted with a commercial RNA miniprep kit (Zymo Research, R2014) following the manufacturer-recommended protocol. Reverse transcription was performed using a commercial kit (Qiagen, 205311) with the protocol recommended by the manufacturer. PCR was performed with a commercial kit (Life Technologies, 4367659) and measured using the Mx3005P qPCR system. A no-RT control experiment was performed to confirm that no detectable DNA was present. Melting curve analysis confirmed that the qPCR product was correct.

### Reporting Summary

Further information on research design is available in the Nature Research Reporting Summary linked to this article.

## Supplementary information

Supplementary Information Supplementary figures.

Reporting Summary

Supplementary Data Supplementary Tables 1–11, containing DNA and RNA sequence information.

## Data Availability

The main data supporting the results of this study are included within the paper and its Supplementary information. The raw datasets generated and analysed during the study are too large to be publicly shared, yet they are available from the corresponding author on reasonable request.
