# Supplementary material for: Multi-arm RNA junctions encoding molecular logic unconstrained by input sequence for versatile cell-free diagnostics
Source: Nat Biomed Eng. 2022 Mar 14;6(3):298–309. doi: 10.1038/s41551-022-00857-7 (PMC8940621; doi:10.1038/s41551-022-00857-7)
Supplement: Supplementary file 1 — Supplementary figures. [file 41551_2022_857_MOESM1_ESM.pdf]

nature portfolio

https://doi.org/10.1038/s41551-022-00857-7

Supplementary information

Multi-arm RNA junctions encoding molecular logic unconstrained by input sequence for versatile cell-free diagnostics

In the format provided by the authors and unedited

Contents

Supplementary Fig. 1 | ON- and OFF-state signals of all 24 LIRAs
Supplementary Fig. 2 | Testing of LIRAs with different loop sizes
Supplementary Fig. 3 | Testing of LIRAs with different input RNA lengths
Supplementary Fig. 4 | RT-qPCR data for LIRA H01 compared to first-generation toehold switch #1
Supplementary Fig. 5 | Representative flow cytometry gating data

Additional Supplementary Information

Supplementary tables containing the sequence information of the RNAs and DNAs used in this study are provided as a spreadsheet file. This file contains the following supplementary tables:

Supplementary Table 1 | Universal primers for plasmid construction
Supplementary Table 2 | Sequences of the Library of 24 LIRAs
Supplementary Table 3 | Sequences of LIRA variants with different loop lengths
Supplementary Table 4 | Sequences of truncated LIRA input RNAs
Supplementary Table 5 | Sequences of LIRA H01 clamp variants
Supplementary Table 6 | Sequences of mRNA-responsive LIRAs
Supplementary Table 7 | Sequences of multi-arm RNA junction logic systems tested in *E. coli*
Supplementary Table 8 | Sequences of pathogen-detecting LIRAs and NASBA primers
Supplementary Table 9 | Sequences of virus-detecting multi-arm RNA junction logic systems and NASBA primers
Supplementary Table 10 | Sequences of multi-arm RNA junction logic systems for influenza subtyping
Supplementary Table 11 | RT-qPCR primers for GFP and 16S rRNA

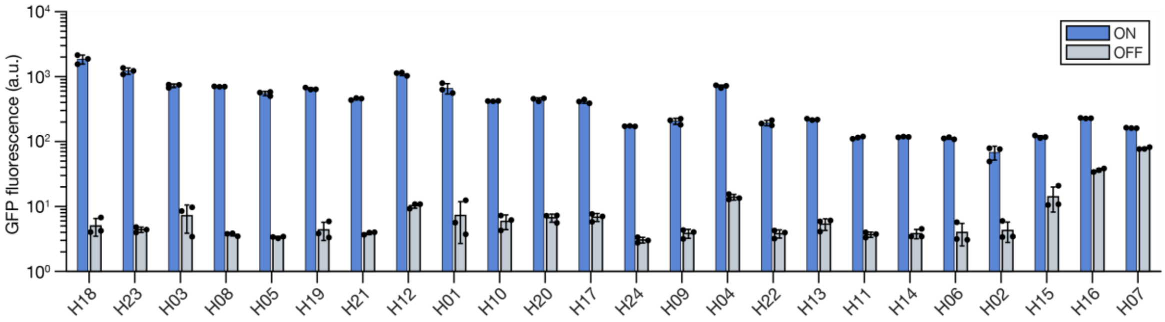

Supplementary Fig. 1 | ON- and OFF-state signals of all 24 LIRAs. Measurements were taken 3 hours after induction with IPTG, *n* = 3 biological replicates, bars represent the geometric mean ± s.d.

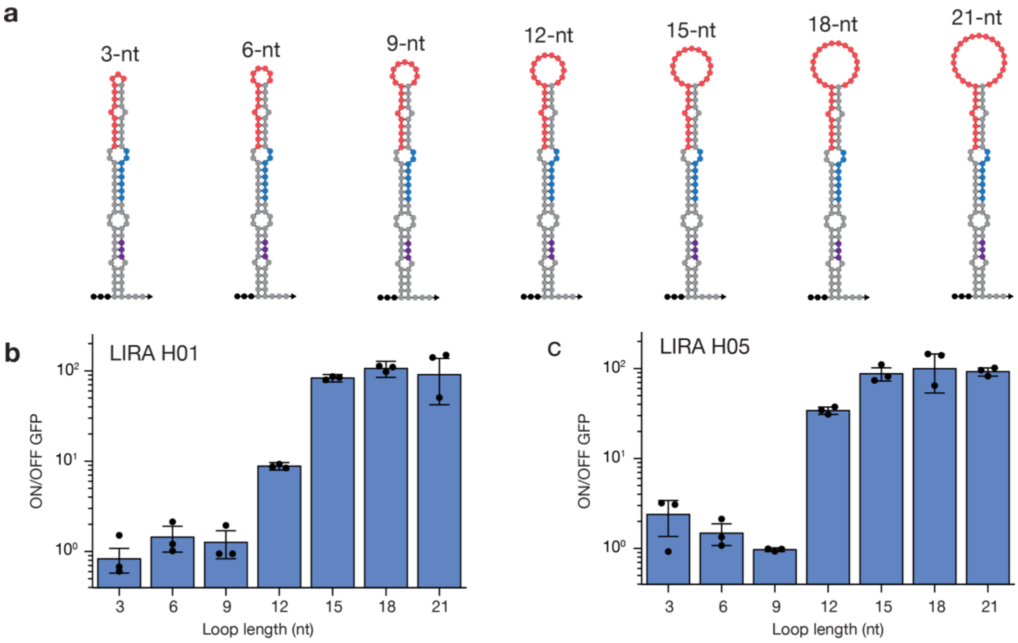

Supplementary Fig. 2 | Testing of LIRAs with different loop sizes. a, Schematic of LIRAs with different loop lengths. **b**, ON/OFF GFP fluorescence ratios of LIRA H01 loop variants tested in *E. coli* with and without expression of the cognate input RNA. **c**, ON/OFF fluorescence ratios of LIRA H05 loop variants. For both sets of systems, device ON/OFF ratios saturate as loop size increases beyond 15 nt. n = 3 biological replicates, bars represent the arithmetic mean ± s.d. for **b** and **c**.

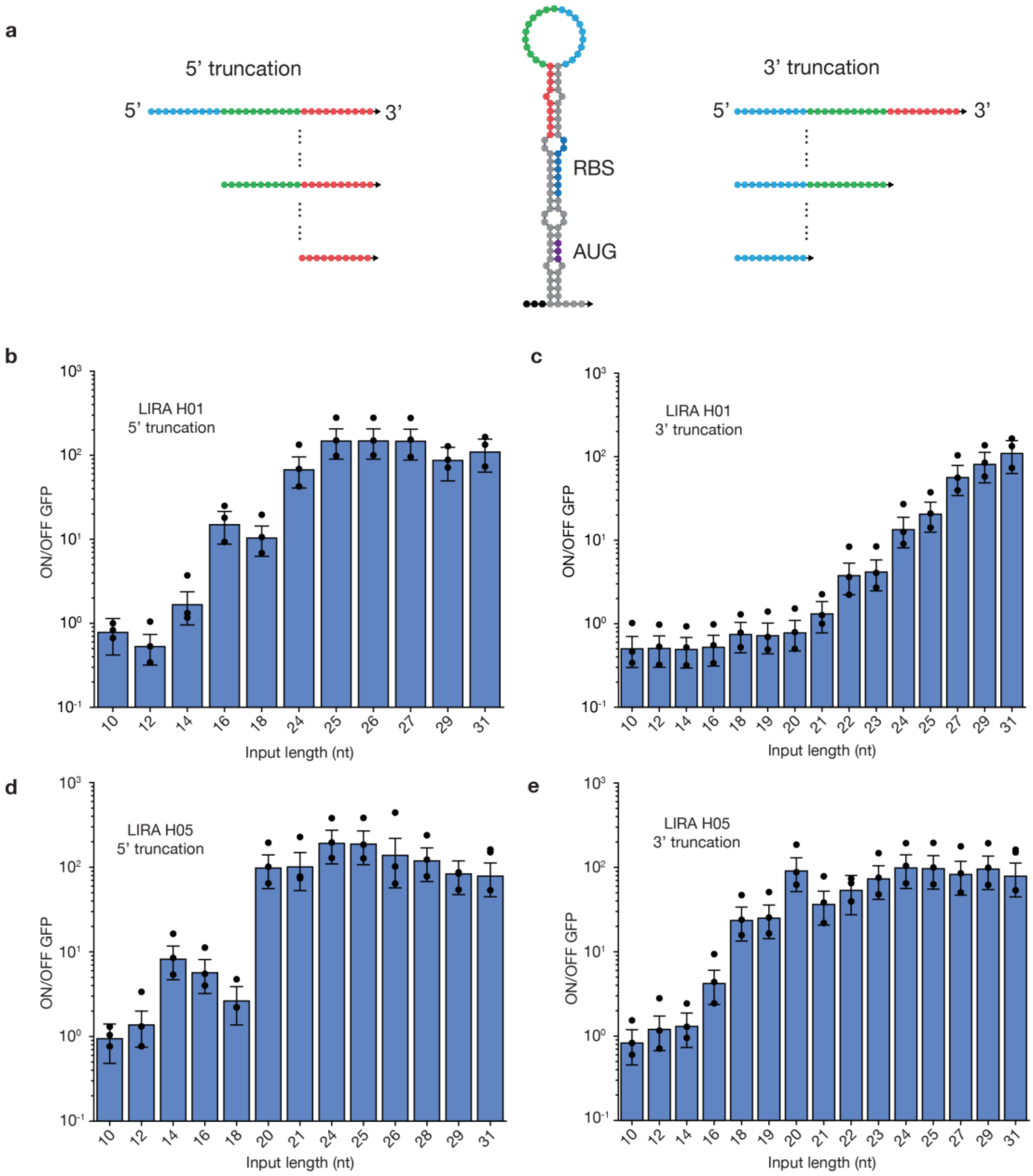

Supplementary Fig. 3 | Testing of LIRAs with different input RNA lengths. a, Schematic of input RNAs truncated from the 5' and 3' ends. **b**, ON/OFF GFP fluorescence ratios of LIRA H01 with inputs truncated from the 5' end. **c**, ON/OFF GFP fluorescence ratios of LIRA H01 with inputs truncated from the 3' end. **d**, ON/OFF GFP fluorescence ratios of LIRA H05 with inputs truncated from the 5' end. **e**, ON/OFF GFP fluorescence ratios of LIRA H05 with inputs truncated from the 3' end. An input RNA length of 31 nt provides the best ON/OFF ratios overall, but shorter inputs can also achieve robust translation activation. *n* = 3 biological replicates, bars represent the arithmetic mean ± s.d. for **b**, **c**, **d** and **e**.

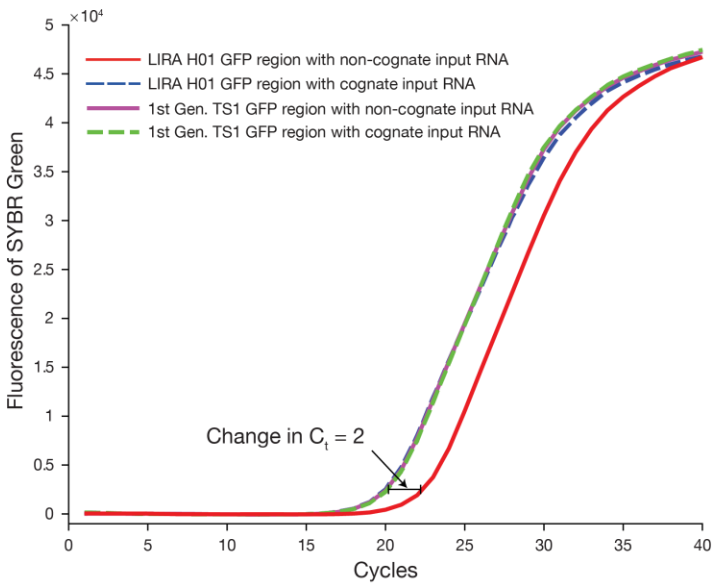

Supplementary Fig. 4 | RT-qPCR data for LIRA H01 compared to first-generation toehold switch #1. RT-qPCR measurements were performed on the *GFP* gene downstream of the riboregulator hairpins. In absence of a cognate input RNA, the C_t value for the LIRA H01 mRNA decreased by two compared to the LIRA H01 mRNA with the cognate input RNA. This result indicates a 4-fold decrease in LIRA H01 mRNA levels without the input RNA and the presence of transcriptional regulation. In comparison, mRNA levels for toehold switch #1 were unaffected by the expression of the input or trigger RNA. For normalization, RT-qPCR was also performed on the housekeeping 16S rRNA and showed the same C_t value across all samples.

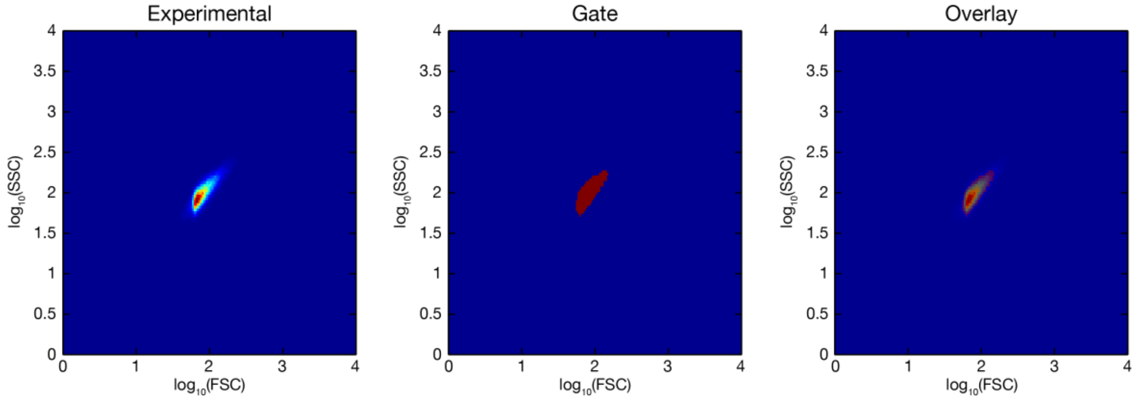

Supplementary Fig. 5 | Representative flow cytometry gating data. Two-dimensional histograms of *E. coli* cells expressing LIRA H11 and its cognate input RNA along the side scatter (SSC) and forward scatter (FSC) channels. A gate was defined based on the SSC and FSC histograms as shown in red (middle histogram) to remove data caused by debris and doublets. Right histogram shows the overlay of the gate over the experimental data.
