# Supplementary material for: Multi-arm RNA junctions encoding molecular logic unconstrained by input sequence for versatile cell-free diagnostics
Source: Nat Biomed Eng. 2022 Mar 14;6(3):298–309. doi: 10.1038/s41551-022-00857-7 (PMC8940621; doi:10.1038/s41551-022-00857-7)
Supplement: Supplementary file 2 — Reporting Summary [file 41551_2022_857_MOESM2_ESM.pdf]

nature research

Corresponding author(s): Alexander A. Green

Last updated by author(s): May 13, 2021

Reporting Summary

Nature Research wishes to improve the reproducibility of the work that we publish. This form provides structure for consistency and transparency in reporting. For further information on Nature Research policies, see our Editorial Policies and the Editorial Policy Checklist.

Statistics

For all statistical analyses, confirm that the following items are present in the figure legend, table legend, main text, or Methods section.

n/a	Confirmed	
☐	☒	The exact sample size (*n*) for each experimental group/condition, given as a discrete number and unit of measurement
☐	☒	A statement on whether measurements were taken from distinct samples or whether the same sample was measured repeatedly
☐	☒	The statistical test(s) used AND whether they are one- or two-sided<br>*Only common tests should be described solely by name; describe more complex techniques in the Methods section.*
☒	☐	A description of all covariates tested
☒	☐	A description of any assumptions or corrections, such as tests of normality and adjustment for multiple comparisons
☐	☒	A full description of the statistical parameters including central tendency (e.g. means) or other basic estimates (e.g. regression coefficient) AND variation (e.g. standard deviation) or associated estimates of uncertainty (e.g. confidence intervals)
☐	☒	For null hypothesis testing, the test statistic (e.g. *F*, *t*, *r*) with confidence intervals, effect sizes, degrees of freedom and *P* value noted<br>*Give P values as exact values whenever suitable.*
☒	☐	For Bayesian analysis, information on the choice of priors and Markov chain Monte Carlo settings
☒	☐	For hierarchical and complex designs, identification of the appropriate level for tests and full reporting of outcomes
☒	☐	Estimates of effect sizes (e.g. Cohen's *d*, Pearson's *r*), indicating how they were calculated

Our web collection on statistics for biologists contains articles on many of the points above.

Software and code

Policy information about availability of computer code

Data collection	CellCapTure (version 4) was used for collecting flow-cytometry data.
Data analysis	Matlab R2017b and Matlab R2019b were used for data analysis.

For manuscripts utilizing custom algorithms or software that are central to the research but not yet described in published literature, software must be made available to editors and reviewers. We strongly encourage code deposition in a community repository (e.g. GitHub). See the Nature Research guidelines for submitting code & software for further information.

Data

Policy information about availability of data

All manuscripts must include a data availability statement. This statement should provide the following information, where applicable:

- Accession codes, unique identifiers, or web links for publicly available datasets
- A list of figures that have associated raw data
- A description of any restrictions on data availability

The main data supporting the results of this study are included within the paper and its Supplementary information. The raw datasets generated and analysed during the study are too large to be publicly shared, yet they are available from the corresponding author on reasonable request.

Field-specific reporting

Please select the one below that is the best fit for your research. If you are not sure, read the appropriate sections before making your selection.

☒ Life sciences ☐ Behavioural & social sciences ☐ Ecological, evolutionary & environmental sciences

For a reference copy of the document with all sections, see nature.com/documents/nr-reporting-summary-flat.pdf

Life sciences study design

All studies must disclose on these points even when the disclosure is negative.

Sample size	Flow cytometry was performed on populations of at least 40,000 cells. Three biological replicates were analysed for each condition. Sample sizes were chosen on the basis of common experimental procedures in microbiology.
Data exclusions	No data were excluded from the analyses.
Replication	At least three biological replicates were measured for each experiment. All attempts at replication were successful and confirmed device function.
Randomization	Organisms were transformed with different DNA and subject to different inducers as necessary, as described in the paper. Any covariates were controlled by processing experimental groups to be compared in identical conditions.
Blinding	The investigators were not blinded to group allocation. Blinding was not possible because the investigators generated the different experimental groups studied.

Reporting for specific materials, systems and methods

We require information from authors about some types of materials, experimental systems and methods used in many studies. Here, indicate whether each material, system or method listed is relevant to your study. If you are not sure if a list item applies to your research, read the appropriate section before selecting a response.

Materials & experimental systems

n/a	Involved in the study	
☒	☐	Antibodies
☒	☐	Eukaryotic cell lines
☒	☐	Palaeontology and archaeology
☒	☐	Animals and other organisms
☒	☐	Human research participants
☒	☐	Clinical data
☒	☐	Dual use research of concern

Methods

n/a	Involved in the study	
☒	☐	ChIP-seq
☐	☒	Flow cytometry
☒	☐	MRI-based neuroimaging

Flow Cytometry

Plots

Confirm that:

☒ The axis labels state the marker and fluorochrome used (e.g. CD4-FITC).

☒ The axis scales are clearly visible. Include numbers along axes only for bottom left plot of group (a 'group' is an analysis of identical markers).

☒ All plots are contour plots with outliers or pseudocolor plots.

☒ A numerical value for number of cells or percentage (with statistics) is provided.

Methodology

Sample preparation	Flow cytometry was performed using a S1000 cell analyzer (Stratedigm) equipped with a high-throughput auto sampler (A600, Stratedigm). Before taking measurements, cells were diluted ~10-fold into phosphate buffered saline (PBS) in 384-well plates. Forward scatter (FSC) was used for the trigger, and ~40,000 individual cells were recorded.
Instrument	Stratedigm S1300EXi
Software	Flow-cytometry data were collected using proprietary software packages from Stratedigm (CellCapTure). The flow-cytometry

Software	data were processed using Matlab.
Cell population abundance	N/A
Gating strategy	Cell populations were gated according to their forward scatter (FSC) and side scatter (SSC) distributions, to remove any doublets and cell debris.

☒ Tick this box to confirm that a figure exemplifying the gating strategy is provided in the Supplementary Information.
